# Quantum exhaustive key search with simplified-DES as a case study

**DOI:** 10.1186/s40064-016-3159-4

**Published:** 2016-09-06

**Authors:** Mishal Almazrooie, Azman Samsudin, Rosni Abdullah, Kussay N. Mutter

**Affiliations:** 1 School of Computer Sciences, Universiti Sains Malaysia (USM), 11800 Minden, Pulau Pinang Malaysia; 2 School of Physics, Universiti Sains Malaysia (USM), 11800 Minden, Pulau Pinang Malaysia

**Keywords:** Quantum cryptanalysis, Grover search, Symmetric cryptography, Block cipher, Quantum simulation

## Abstract

To evaluate the security of a symmetric cryptosystem against any quantum attack, the symmetric algorithm must be first implemented on a quantum platform. In this study, a quantum implementation of a classical block cipher is presented. A quantum circuit for a classical block cipher of a polynomial size of quantum gates is proposed. The entire work has been tested on a quantum mechanics simulator called libquantum. First, the functionality of the proposed quantum cipher is verified and the experimental results are compared with those of the original classical version. Then, quantum attacks are conducted by using Grover’s algorithm to recover the secret key. The proposed quantum cipher is used as a black box for the quantum search. The quantum oracle is then queried over the produced ciphertext to mark the quantum state, which consists of plaintext and key qubits. The experimental results show that for a key of *n*-bit size and key space of *N* such that $N=2^n$, the key can be recovered in $\mathcal {O} \left(\frac{\pi }{4}\sqrt{N} \right)$ computational steps.

## Background

Information security heavily relies on modern cryptography. Most of the cryptographic algorithms are designed to be resistant against attacks. Asymmetric cryptography or public-key cryptography is one of the cryptographic primitives based on computationally hard problems. For instance, the RSA algorithm (Rivest et al. 1978) in asymmetric cryptography, a large integer number *N* of more than 300 digits is given, and the task is to factorize *N* to its product of two big prime numbers *p* and *q*. This computationally hard problem, which RSA is based on, is called factoring problem, which protects the system from attacks by adversaries. Using General Number Field Sieve (GNFS) algorithm in asymptotic time of $\mathcal {O} \left(exp \left(\left(\frac{64}{9}b \right)^\frac{1}{2}(\log b)^\frac{2}{3} \right)\right)$ (Wiener 1990), that can factor large integers, is the most efficient attack on a classical computer. Although asymmetric cryptosystems that are based on hard problems have been proven secure, they are not efficient for the use in real-time encryption of large messages. Thus, one of the main uses of RSA is to distribute the secret key shared by two parties that are communicating in a secure channel; in this task, the second primitive of cryptography (symmetric cryptography or private-key cryptography) performs the real-time encryption.

In symmetric cryptography, when the symmetric cryptosystem exhibits a good randomness level and the exhaustive search for the secret key is the only attack that can break the cryptosystem, the hardness or strength of the cryptosystem is determined by the size of the encryption key. A key with *n* bits size has $2^n$ possibilities of keys and therefore $\mathcal {O}(2^n)$ steps are needed to try all of these possibilities. For example, $2^{128}$ operations are required to try all the possibilities of a 128-bit key, which cannot be achieved using conventional or classical computing techniques. Advanced Encryption Standard (AES; Stallings 2002) and Data Encryption Standard (DES; Coppersmith et al. 1997) are well-known symmetric cryptographic algorithms.

Asymmetric and symmetric cryptography are believed to be secure against any attack using classical computers. Unfortunately, this view is no longer valid in the present of the quantum mechanics where the calculations are performed based on the behavior of particles at subatomic levels. Thus, quantum computing poses threats to asymmetric and symmetric cryptography. Regarding asymmetric cryptography, in the presence of scalable quantum computers, the cryptographic algorithm based on the factoring problem would be completely jeopardized (Shor 1997). Various studies have been published on quantum number factorization (Lanyon et al. 2007; Markov and Saeedi 2013; Martín-López et al. 2012; Lucero et al. 2012). Consequently, other alternative solutions besides the factoring problem are investigated, such as code-based cryptography and lattice-based cryptography (Bernstein et al. 2008). Moreover, some solutions to the key distribution problem have come from quantum mechanics and opened the field of quantum cryptography (Nicolas et al. 2002; Cláudio and Viana 2010; Mihara 2007; Jeong and Kim 2015).

In the scope of this study concerning symmetric cryptography, the situation remains doubtful compared with the clear impact of quantum computing on asymmetric cryptography. The only known and clear quantum threat to symmetric algorithms is that the exhaustive key search can be performed more efficiently on the quantum platform with quadratic speedup using Grover’s algorithm (Grover 1996). However, the quantum exhaustive search attack cannot be applied unless the symmetric algorithm is implemented on the quantum platform. Few studies have been published on quantum symmetric cryptanalysis whereas a large number of studies has focused on asymmetric cryptography.

One of the first papers on quantum cryptanalysis of block ciphers is by Akihiro (2000), who discussed the effect of Grover’s algorithm when used to recover the secret key of block ciphers based on the assumption that the block cipher was already implemented on quantum and used the block cipher as a black box for Grover’s algorithm. The researchers discussed that the security of a block cipher could be evaluated by using Prassarad, Høyer, and Tapp’s quantum algorithm (Brassard et al. 1998).

Roetteler et al. (2015) published a note on quantum-related key attacks based on three assumptions: the secret key can be found with a small number of plaintext/ciphertext pairs, the block cipher can be implemented efficiently as a quantum circuit, and the related keys can be queried in superpositions. The researchers stated that even though the attack is powerful, it is unlikely to pose a practical threat because of the difficulties in querying the secret keys in superpositions.

In quantum asymmetric cryptanalysis such as RSA, when factoring an integer number *N* into its two prime numbers *p* and *q*, implementing the RSA algorithm on a quantum platform is unnecessary. By contrast, when applying a quantum attack on a symmetric cipher to determine the secret key, the cipher algorithm must be first implemented on a quantum platform. We claim that this is one of the main reasons for the small number of published papers on quantum symmetric cryptography compared with asymmetric cryptography. Moreover, the few published studies are based on the assumption that the symmetric cryptosystem algorithm is implemented efficiently on a quantum platform. In this study, a quantum circuit for a classical symmetric cryptosystem is introduced.

This paper is organized as follows: the simplified-DES cryptosystem is introduced in second section. A preview on Grover’s algorithm is presented on third section. The proposed quantum circuit is explained in detail in fourth section. The complexity analysis is conducted in fifth section. The experimental results are presented and discussed in sixth section. Finally, seventh section provides the conclusion and suggestions for future research.

## Simplified-DES

**Fig. 1:**
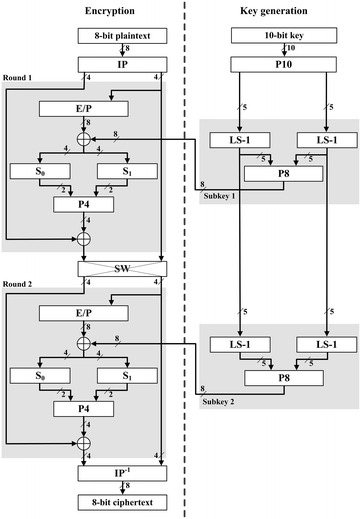
Simplified-DES

Simplified-DES (SDES) is a simple version of the well-known cipher DES developed by Schaefer (1996). With small parameters, SDES has similar properties and structure to DES (Stallings 2010). The small structure of SDES represents accurately the structure of the original DES. Subsequently, SDES is a good case study to represent Feistel class block ciphers. It is highly likely that if SDES can be coded into a quantum circuit, then a good number of Feistel class block ciphers can be coded into quantum circuit as well. The SDES algorithm consists of key generation and encryption function $f_k$ as shown in Fig. 1.

**Fig. 2:**
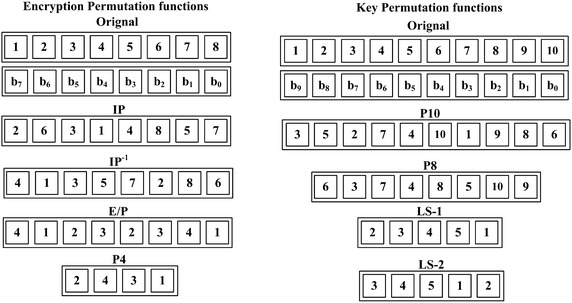
Permutation functions in simplified-DES

In the key generation of SDES, two 8-bit subkeys are generated from the main 10-bit secret key. First, the key is permuted through P10. Then, the 10-bit key is divided into two halves, each with 5 bits. The one-bit left shift (LS-1) is applied to each half and the output after the left shifting is combined again. Then, the 10-bit output goes through the permutation function P8 to generate the first subkey $k_1$. The combined output after (LS-1) is separated again and left shifted by two bits through (LS-2). Thereafter, the output goes through function P8 to produce the second subkey $k_2$. All of the permutation functions are illustrated in Fig. 2.

The SDES encryption algorithm, as shown in Fig. 1, has only two rounds of encryption. First, 8-bit plaintext is permuted through the initial permutation function IP. Then, the plaintext is divided into two halves. The right half of the plaintext is expanded to 8 bits by applying the expansion function E/P. Thereafter, the output from E/P is XOR-ed with the first subkey. The 8-bit output is then divided into two 4-bit halves. The left half is fed to the substitution box (S-box) S_0_ and the right half goes to S_1_. The S-boxes S_0_ and S_1_ are the most complicated components of the SDES algorithm. One S-box can be represented as a $4 \times 4$ matrix. The first and fourth bits of the input are considered as a 2-bit number used to specify the row of the S-box. The second and the third bits of the input specify the column of the S-box. The two S-boxes S_0_ and S_1_ are represented as follows:

$$\begin{aligned} S_0 = \begin{bmatrix} 1&0&3&2\\ 3&2&1&0\\ 0&2&1&3\\ 3&1&3&2 \end{bmatrix} \quad S_1 = \begin{bmatrix} 0&1&2&3\\2&0&1&3\\3&0&1&0\\2&1&0&3 \end{bmatrix}. \end{aligned}$$

The 4-bit output from S_0_ and S_1_ is XOR-ed with the left half of the plaintext to produce the 4-bit half of the ciphertext. The right 4-bit half of the plaintext is not altered in the first round. The switch function SW interchanges the right and left halves before the second round of SDES takes place. The second round is identical to the first round except that the second subkey $k_2$ was used instead of $k_1$. Finally, the output of the second round is then subjected to the inverse of the initial permutation IP-1 and the ciphertext is produced.

## Grover’s algorithm

This section presents a view of quantum bits (qubits) and the quantum search algorithm (Grover’s algorithm). As a reference, quantum information and unitary transformation are discussed in quantum computing introductory books such as David Mermin (2007).

The quantum bit (qubit) is characterized by two orthogonal states $\vert 0 \rangle$ and $\vert 1 \rangle$. In contrast to classical bit, the qubit can be in a superposition state as follows:

1 $$\begin{aligned} \large \vert \psi \rangle =\alpha \vert 0 \rangle +\beta \vert 1 \rangle \end{aligned}$$

where $\alpha$ and $\beta \, \epsilon \,\mathbb {C}$, which representing the amplitude probability such that $|\alpha |^2+|\beta |^2=1$. Those states of the qubit can be expressed as vectors in two-dimensional Hilbert space $\mathcal {H}$ as:

2 $$\begin{aligned} \vert 0 \rangle =\begin{pmatrix} 1 \\ 0 \end{pmatrix},\, \vert 1 \rangle =\begin{pmatrix} 0 \\ 1 \end{pmatrix},\,\vert \psi \rangle =\begin{pmatrix} \alpha \\ \beta \end{pmatrix} \end{aligned}$$

The quantum search algorithm was discovered by Grover (1996) and named after him. Grover’s search algorithm and Shor’s period finding algorithm (Shor 1997), along with their extensions, constitute the masterpiece algorithms of quantum computations (David Mermin 2007).

### *Problem definition*

 Given an unstructured database of *N* elements, find the element $a \in N$. This can be modeled as a function $f:\{0,1\}^n\rightarrow \{0,1\}$, where the space $N = 2^n$ , for any $x \in \{0,1\}^n$:

3 $$\begin{aligned} f(x) = \left\{ \begin{array}{ll} 1 &\text{if } x = a \hbox{(a solution)};\\ 0 &\text{otherwise } \text{(not } \text{a } \text{ solution). }\end{array} \right. \end{aligned}$$

When the database is unstructured, the element ‘*a*’ can be found among *N* random elements (by assuming the uniform probability distribution) with probability of 1 / *N*. Therefore, on a classical computer, $\mathcal {O}(N) = \mathcal {O}(2^n)$ steps are needed to find ‘*a*’.

On the other hand, quantum computing using Grover’s algorithm, the element ‘*a*’ can be found with a significant speedup that is quadratically faster than that on any classical computer. The search through an unstructured database can be accomplished within $\mathcal {O}(\sqrt{N})$ computational steps (Boyer et al. 1998; Christof 1999). The procedure of Grover’s algorithm is shown in Algorithm 1. 
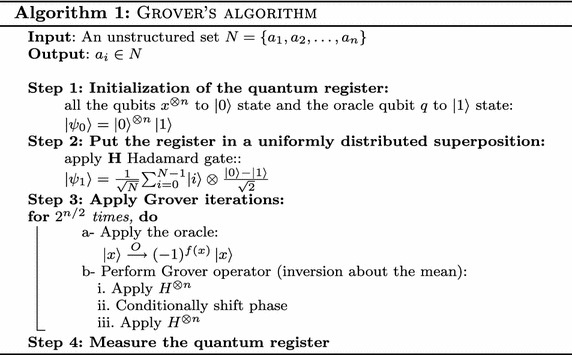


Additional discussions with circuit illustration on the oracle and the inversion about the mean are conducted in fourth section. All the steps of Grover’s algorithm listed in Algorithm 1, can be expressed as follows:

4 $$\begin{aligned} \large ((2\vert \psi \rangle \langle {\psi }|-I)\mathbf {O})^R \end{aligned}$$

where $\mathbf {O}$ is the oracle and *R* is the number of iterations. Assume there is a function $f:\{0,1\}^n\rightarrow \{0,1\}$ has a unique solution $i \in \{0,1\}^n$, and $N=2^n$, the number of iterations *R* in Eq. 4 is calculated as follows:

5 $$\begin{aligned} R=\frac{\pi }{4}\sqrt{N}. \end{aligned}$$

In the case when there are multiple solutions (*M*), *R* is calculated as follows:

6 $$\begin{aligned} R=\frac{\pi }{4}\sqrt{\frac{N}{M}}\, . \end{aligned}$$

## SDES quantum circuit

**Fig. 3:**
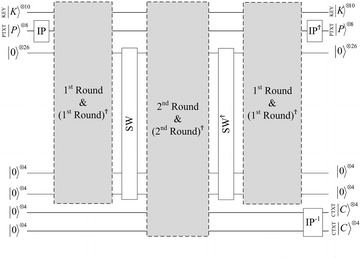
The proposed quantum SDES (QSDES)

**Fig. 4:**
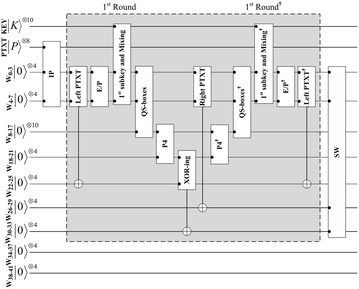
The circuit of the first encryption round

The proposed quantum circuit of the cipher SDES is shown in Fig. 3. The encryption key is composed of ten qubits and another eight qubits defined for the plaintext. Eight ancilla qubits can be used for the ciphertext. More ancilla qubits are used for the work space to design the quantum SDES circuit which we named Quantum SDES (QSDES). Figure 4 illustrates the steps of the first encryption round. In the following subsections, each part of the circuit is discussed in detail.

### Initial permutation and expansion

**Fig. 5:**
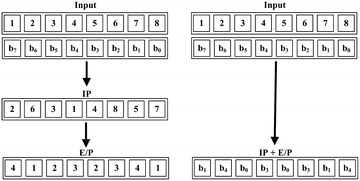
Integrating IP and E/P in one step

In classical computing, the permutation process can be achieved using temporary variables and then the data can be copied to those temporary variables by changing the indices. In quantum, fan-out circuit is a good solution to perform quantum permutation over the qubits. The powerful fan-out circuit has been studied in detail by Høyer and Ŝpalek (2005). Both of the initial permutation and the expansion of the right half of the plaintext are integrated in one step to minimize the number of quantum gates. Integrating these two steps is achieved as illustrated in Fig. 5.

**Fig. 6:**
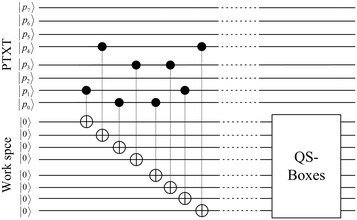
IP and E/P circuit

The quantum permutation and expansion circuit are shown in Fig. 6. The quantum permutation is applied using eight CNOT gates and eight ancilla qubits. The left half of the plaintext is copied using the fan-out circuit to other ancilla qubits, and then later XOR-ed with the output of the S-boxes. In fact, this step can be ignored and more ancilla qubits can be saved; however, for the benefit of the reader, we try to facilitate the comparison of the quantum circuit QSDES with the classical SDES.

### First subkey generation and key mixing

**Fig. 7:**
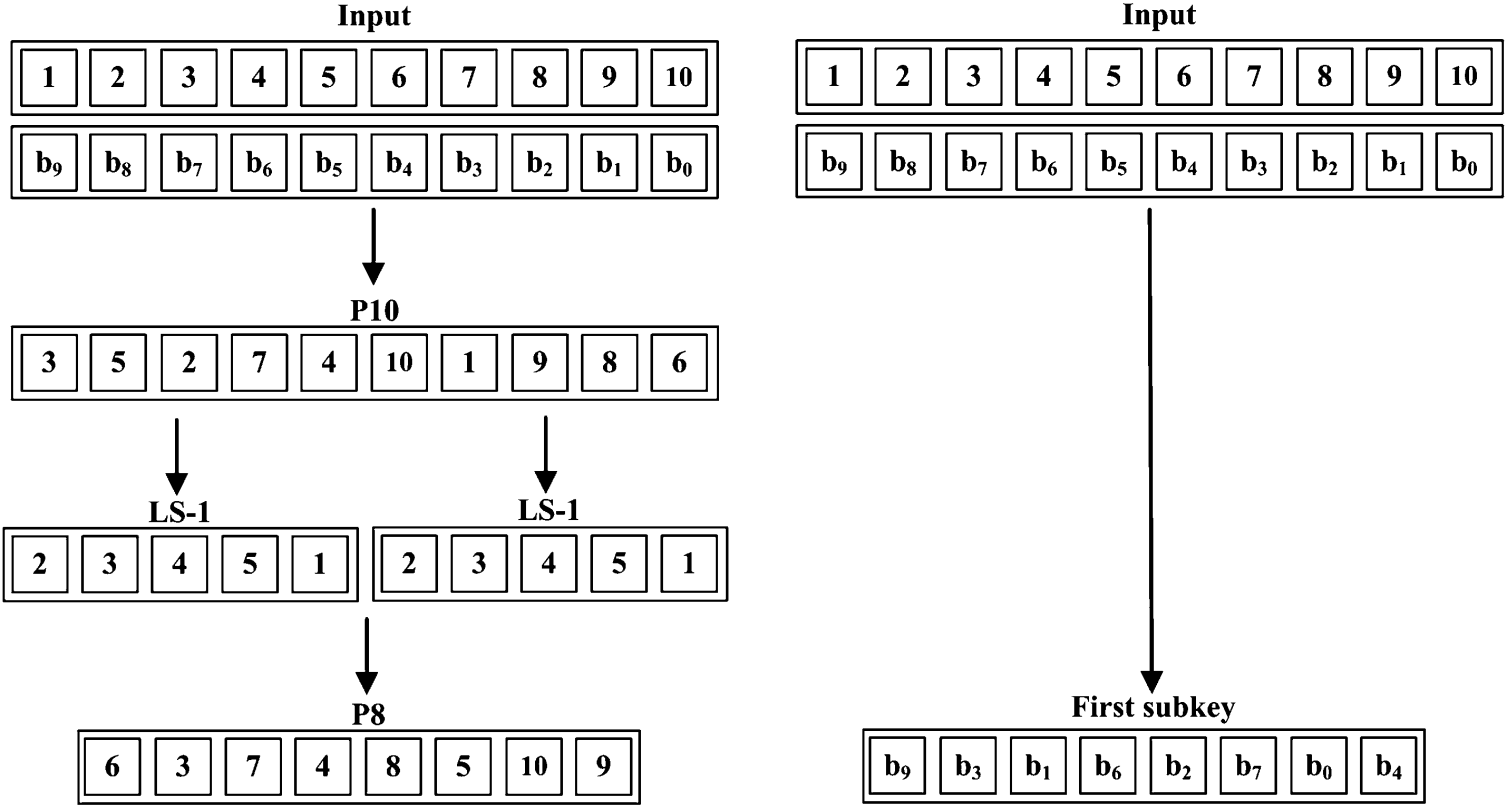
Integrating first subkey permutations into one single step

Similar to DES, subkey generation of SDES involves a group of bit permutations over the secret key. Even the left shift rotations can be considered as permutations. Regarding the first subkey, the different permutation steps, namely, P10, LS-1, LS-1, and P8, are integrated into one step in a similar way as shown in “Initial permutation and expansion” section. Figure 7 shows how the first subkey is generated in one step. Then, the generated subkey $k_1$ is XOR-ed with the expanded plaintext using 8 CNOT gates.

### The quantum substitution boxes

The quantum S-boxes (QS-boxes) are the most complicated parts of the entire circuit of QSDES and they require a larger number of quantum gates. The quantum gates are still considered to be a polynomial circuits, as discussed in the complexity analysis section. In general, S-boxes are essential components in symmetric algorithm because they satisfy the Shannon property of confusion (Shannon 1949). The confusion property hides the relation between the secret key and the ciphertext; this property has to be achieved even in the quantum platform when the key is in a superposition.

The S-boxes can be categorized into two types: statistically defined S-boxes and dynamically key-dependent generated S-boxes. Moreover, the statistically defined S-boxes can be generated dynamically by different methods such as hand crafted, mathematically generated data dependent, etc. (Stallings 2002). Concerning memory space, the S-boxes can be generated dynamically at the run time or can be predefined statistically. Conversely, the key-dependent dynamically generated S-boxes, such as Blowfish (Schneier 1993) and Twofish (Schneier et al. 1999) ciphers, as well as the elements of the S-boxes, continue changing in accordance with the secret key.

In the case of SDES, the S-boxes are predefined statistically. In the following context, the design of the Quantum S-box (QS_0_) is discussed in details while the second quantum S-box (QS_1_) is omitted as the only difference is in the values of the elements of the S-box. The table of QS_0_ which shown in Table 1, is rewritten as a lookup table derived from the matrix of Eq. 2.

**Table 1:** QS_0_ lookup table

Input	0010	0111	1000	0000	0101	1011	1100	0011	0110	1010	1111	0001	0100	1001	1101	1110
Output	00	00	00	01	01	01	01	10	10	10	10	11	11	11	11	11

**Fig. 8:**
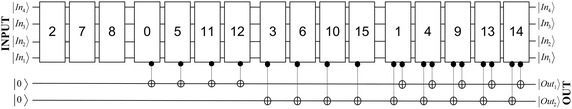
QS_0_ circuit as illustrated in Table 1

As shown in Table 1, the 16 possible inputs of the 4-bit input are listed with the corresponding 2-bit output of each input. The quantum circuit of QS_0_ is presented in Fig. 8. As the output of QS_0_ is two qubits, one of the four states 00, 01, 10, and 11 could be expected or all of these four states could be the output simultaneously with equal probabilities. In the circuit shown in Fig. 8, the first top four qubits are the input of QS_0_. Then, three ancilla qubits are needed for the work space and two qubits for the output.

**Fig. 9:**
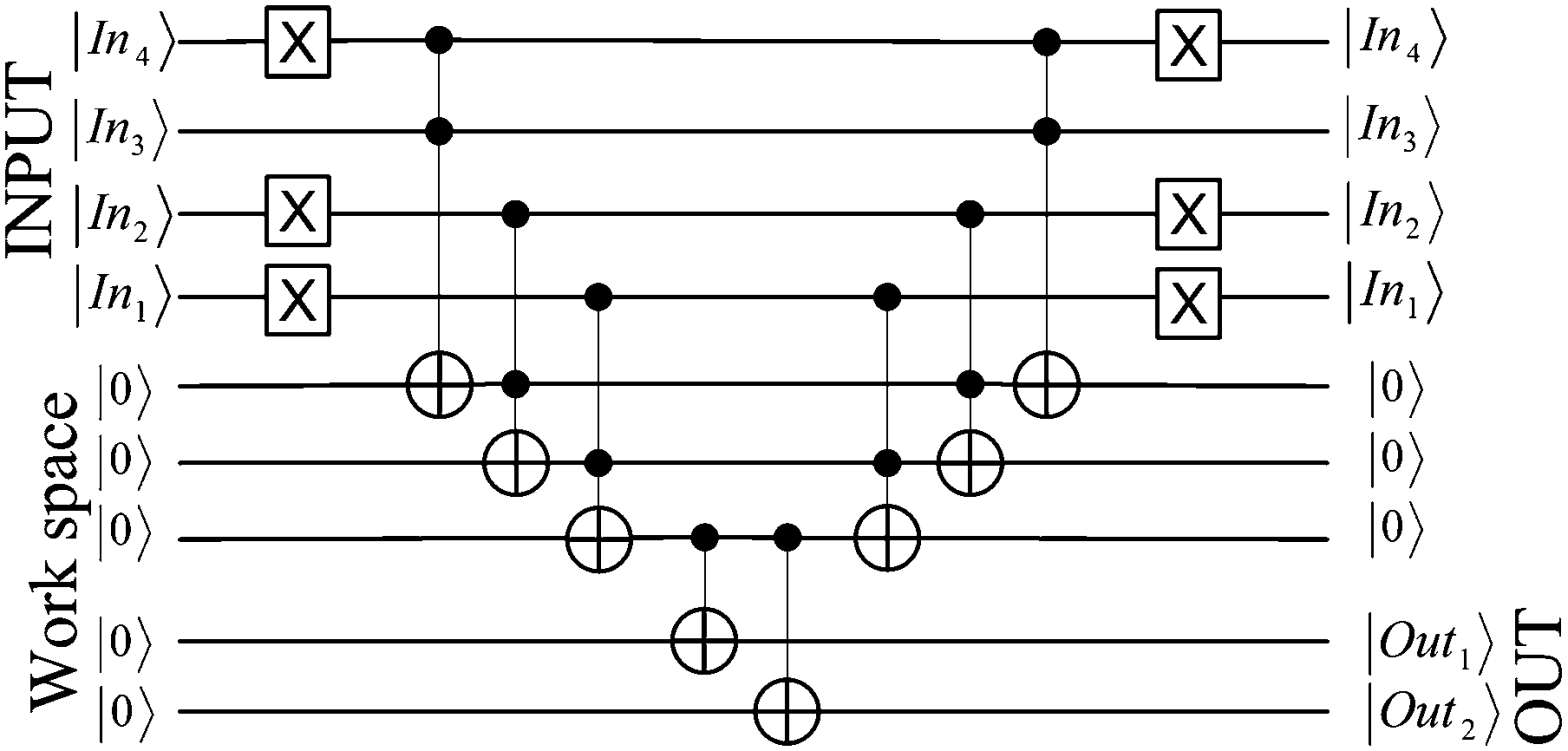
Circuit of the state 4 of S_0_

The circuit, when the input is 4 and the output state is 11, is detailed in Fig. 9. First, the binary representation of 4 (0100) is implemented using Pauli X gates to represent 0. Thereafter, three Toffoli gates are used to compose the Boolean circuit. The ancilla qubits $Out_1$ and $Out_2$ are triggered to the state 1 if the input to QS_0_ is 4. According to the lookup table (Table 1), the input 4 provides the output 11; therefore, the ancilla qubits $Out_1$ and $Out_2$ need to be triggered using two CNOT gates as shown in the circuit. Thereafter, the three Toffoli gates are applied again to reverse the process.

### XOR-ing the right half of the plaintext

The output four qubits from QS_0_ and QS_1_ are permuted through P4 as in the original classical algorithm. The output after the quantum permutation of P4, is XOR-ed with the right half of the plaintext by using four CNOT gates. P4 is performed in a similar way as in the previous subsections. All of the steps in the previous subsections, from plaintext expansion to the last process, are reversed, as shown in Fig. 4. In this proposed design, no garbage qubits hold states. All of the ancilla qubits will be reused in the next encryption round. Therefore, those qubits must be returned to their initial states.

### The switch function

The first round of SDES alters the left half of the plaintext, whereas the right half is untouched. The switching function is constructed using four quantum SWAP gates to interchange the four qubits on the left with the four qubits on the right. A quantum SWAP gate can be constructed from three CNOT gates, which means that 12 CNOT gates are needed for the switch function.

### The second encryption round

Because of the reversal process, all of the work space ancilla qubits are set to their initial states, which make them reusable for the second round of encryption. Only ancilla qubits that hold the produced ciphertext of the first round cannot be used. The second encryption round is performed similarly to the first round. It takes the input qubits after SW and produces the output ciphertext in the last ancilla qubits. In contrast to the first round, no IP involved in this round; thus, the round starts with plaintext expansion function E/P.

The last function in classical SDES is the permutation function IP$^{-1}$, which is the inverse of the IP function. This function is integrated within the second round in the same way as the IP is integrated in the first round. Finally, all the steps involved in this round are inversed, as shown in Fig. 3. For instance, the key qubits are $\vert K \rangle ^{\otimes 10}$, the plaintext qubits are $\vert P \rangle ^{\otimes 8}$, and the ciphertext are in the last ancilla qubits $\vert C \rangle ^{\otimes 4}$ and $\vert C \rangle ^{\otimes 4}$.

### Black box of quantum search

The QSDES circuit is designed with consideration of the fact that the entire circuit will be used as a black box or Oracle for Grover’s quantum search. Thus, no garbage qubits are involved in the circuit such that for every iteration of Grover’s algorithm, all the qubits return to their initial states, resulting in multiple levels of reversibility in the circuit. The first reversibility level is within the quantum S-boxes where the processes are reversed. The second reversibility level is within every encryption round, and the third level of reversibility is when the complete round is reversed (in case of the first round).

**Fig. 10:**
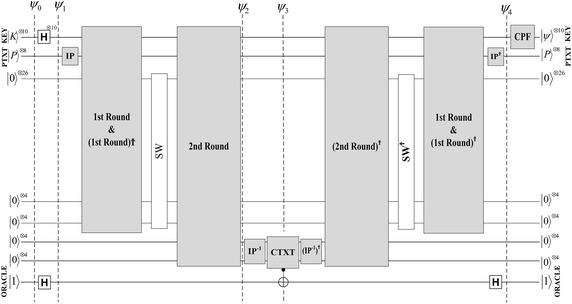
Quantum search for the encryption key of QSDES

Grover’s algorithm, as mentioned in third section, searches for a marked element(s) through many different input states of equal probabilities. In a quantum exhaustive key search attack, the input is a chosen plaintext and its corresponding ciphertext, and the output is the secret key. The complete quantum exhaustive search for the encryption key is shown in Fig. 10.

First, the 10 key qubits $(k_0-k_9)$ are initialized to state 0 and the plaintext qubits $(p_0-p_7)$ are set according to the chosen plaintext. In the circuit shown in Fig. 10, the chosen plain text is (0001 0000). All other ancillas, which are used as work space, are set to 0. One more ancilla qubit is needed as an oracle qubit, which is set to 1 using Pauli X gate. The $\vert \psi _0 \rangle$ phase is at the initialization step and the quantum register is as illustrated by Eq. 7.

7 $$\begin{aligned} \vert \psi _0 \rangle&=\vert K \rangle ^{\otimes 10} \otimes \vert P \rangle ^{\otimes 8} \otimes \vert q \rangle \nonumber \\&=\vert k_9 k_8 k_7 k_6 k_5 k_4 k_3 k_2 k_1 k_0 \rangle \otimes \vert p_7 p_6 p_5 p_4 p_3 p_2 p_1 p_0 \rangle \otimes \vert q \rangle \nonumber \\&=\vert 00 0000 0000 \rangle \otimes \vert 0001 0000 \rangle \otimes \vert 1 \rangle \end{aligned}$$

Hadamard operators H are applied for every key qubit $(k_0-k_9)$. For equivalency, Hadamard gates $H^{\otimes k}$ are applied to create equal superpositions for all possible states of the key. In addition, another Hadamard operator is applied to the oracle qubit. The quantum register at $\vert \psi _1 \rangle$ is shown in Eq. 8.

8 $$\begin{aligned} \vert \psi _1 \rangle&=H\vert K \rangle ^{\otimes 10} \otimes \vert P \rangle ^{\otimes 8} \otimes H\vert q \rangle \nonumber \\&=\frac{1}{\sqrt{K}} \sum _{i=0}^{K-1} \vert k_i \rangle \otimes \vert 0001 0000 \rangle \otimes \frac{\vert 0 \rangle -\vert 1 \rangle }{\sqrt{2}} \nonumber \\&=\frac{1}{\sqrt{2^{10}}} \sum _{i=0}^{2^{10}-1} \vert k_i \rangle \otimes \vert 0001 0000 \rangle \otimes \frac{\vert 0 \rangle -\vert 1 \rangle }{\sqrt{2}} \nonumber \\&=\frac{1}{32} \sum _{i=0}^{1024-1} \vert k_i \rangle \otimes \vert 0001 0000 \rangle \otimes \frac{\vert 0 \rangle -\vert 1 \rangle }{\sqrt{2}} \end{aligned}$$

**Fig. 11:**
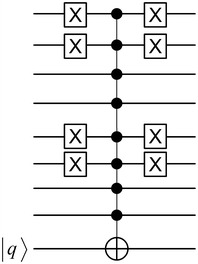
Ciphertext implementation circuit (CTXT)

The chosen ciphertext is implemented in the circuit before the Grover oracle takes place. The corresponding ciphertext of the plaintext (0001 0000) is (0011 0011). The circuit in Fig. 11 illustrates the implementation of the ciphertext.

In classical computing, the SDES algorithm can be expressed as the following:

9 $$\begin{aligned} SDES(K, P) = C \end{aligned}$$

where *K* is the key, *P* is the plaintext, and *C* is the output ciphertext. Similarly, in quantum the QSDES algorithm can be expressed the same way when there is no superposition involved:

10 $$\begin{aligned} QSDES\left( {\mathop{\bigotimes}\limits_{i=0}^{9}}K_i, {\mathop{\bigotimes}\limits_{i=0}^{7}}P_i\right) = {\mathop{\bigotimes}\limits_{i=0}^{7}}C_i \end{aligned}$$

However, when the key is in superposition, all the possible ciphertexts encrypted by all possible 10-qubit keys for the chosen plaintext can be produced at once. Therefore, QSDES with key in superposition can be expressed as follows:

11 $$\begin{aligned} QSDES \left( H \left( {\mathop{\bigotimes}\limits_{i=0}^{9}}K_i \right) , {\mathop{\bigotimes}\limits_{i=0}^{7}} P_i \right) = \sum _{i=0}^{1024-1} \left( {\mathop{\bigotimes}\limits_{i=0}^{7}}C_i \right) \end{aligned}$$

Thus, at phase $\vert \psi _2 \rangle$, all the expected ciphertext generated by all possibilities of the 10-qubit key for the chosen plaintext are produced. In fact, the quantum oracle is applied over the ciphertext, not the key itself. However, according to the oracle answer, the whole quantum state is influenced. Therefore, the oracle shown in Algorithm 1 is rewritten as the following equation:

12 $$\begin{aligned}&\vert \psi _3 \rangle \xrightarrow {O}(-1)^{f(k)}\vert \psi \rangle \nonumber \\&\vert \psi _3 \rangle \xrightarrow {O}(-1)^{QSDES_{(P_i,C_i)}(k)}\vert \psi \rangle \end{aligned}$$

Therefore, once the chosen ciphertext is found, the oracle flips the quantum state that includes the target ciphertext in the quantum register. For instance, the secret key we are looking for is marked at phase $\vert \psi _3 \rangle$. All of the previous steps are reversed and all the qubits in the quantum register are set to their initial values at phase $\vert \psi _4 \rangle$.

**Fig. 12:**
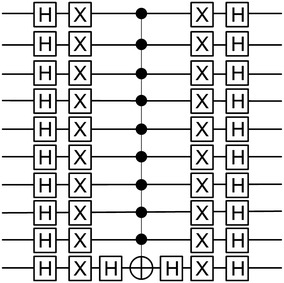
Conditional phase flip circuit (CPF)

Grover operator or the inversion about the mean is also called Conditional Phase Flip (CPF). CPF circuit which shown in Fig. 10, is illustrated in detail in Fig. 12. At this phase, the marked state in the quantum register, which has a different phase from other states, is constructively interfered, whereas all other states in the quantum register are distractively interfered.

## Complexity analysis

The complexity analysis is conducted in term of computing the size of the quantum gates used in the proposed circuit (size of the circuit). The calculations are performed with respect to subkey size ($K_s$), plaintext size ($P_s$), number of rounds ($R_n$), number of permutation functions ($P_n$), input size of S-box ($S_{in}$), and output size of S-box ($S_{out}$). Regarding the key generation process for SDES, since all steps of generating one subkey are integrated in one step then 8 CNOT gates are needed corresponds to the size of the subkey. Since there are two encryption rounds then the number of CNOT gates = $R_n \times K_s$.

The encryption function of QSDES consists of four permutation steps (XOR-ing left half of PTXT, E/P, P4, and XOR-ing the right half of PTXT), key XOR-ing, and two substitution processes (S_0_ and S_1_). The key XOR-ing is already calculated when computed the circuit size of the key generation which is 8 CONT gates. The E/P permutation function needs 8 CNOT gates. Each of the other permutation functions needs 4 CNOT gates corresponds to the half of the plaintext size. Therefore, the circuit size of the permutation functions can be expressed as number of CNOTs = $(P_n \times P_s)/2$.

The largest number of quantum gates used is in the substitution process. Every S-box of QSDES has 16 states corresponds to the size of the input to the S-box which is $2^4$. Each state of them needs X Pauli gates to implement the zeros. Thus, approximately 32 X Pauli gates are needed for the 16 states. In addition, every state needs 3 Toffoli gates and 2 CNOT gates. Therefore, for the 16 states of one S-box, $16 \times 3 = 48$ Toffoli gates and $16 \times 2 = 32$ CNOT gates are used. Thus, the total number of quantum gates needed is:

- number of X Pauli gates = $2^{S_{in}} \times 2 = 16 \times 2$,
- number of Toffoli gates = $2^{S_{in}} \times S_{in} -1 = 16 \times 3$, and
- number of CNOT gates = $2^{S_{in}} \times S_{out} = 16 \times 2$.

The total number of all used gates is then multiplied by 2 for the reversal process within the S-box. In addition, for the reversal process within every round, the total number of gates is multiplied by 2. The conducted complexity analysis provides an evidence that the SDES can be implemented efficiently with a polynomial size of quantum gates. Although the largest number of used gates is in S-box design which is exponentially related to the input size of the S-box but this can be considered as a polynomial since most of the block ciphers have S-boxes of input size of $2^8$ or less such as AES, Blowfish, Towfish, etc.

## Experiments and results

In this section, the quantum simulation used in this study is briefly introduced and the simulation results are interpreted. Then, the functionality of the proposed QSDES is verified and compared with SDES. The quantum exhaustive search results are shown in the last subsection.

### Simulation of quantum mechanics

The C library (libquantum; http://www.libquantum.de/) is used to simulate the QSDES and to apply the quantum search. Libquantum offers high performance and low memory consumption. To interpret the result of libquantum, we present the following values of the quantum register at phase $\vert \psi _0 \rangle$, which is the initialization stage of the circuit in Fig. 10:

13 $$\begin{aligned} \underbrace{(\overbrace{1.000000}^\text {a} + \overbrace{0.000000i)}^\text {b}}_\text {1} \underbrace{\vert 16 \rangle }_\text {2} \underbrace{(1.000000e+00)}_\text {3} \underbrace{\vert \overbrace{0000000000}^\text {a} \overbrace{0001 0000}^\text {b} \rangle }_\text {4} \end{aligned}$$

The preceding results are interpreted as follows:

1. This is the probability amplitude of the states of the quantum register. It is a complex number in Hilbert space. It is also used to calculate the probabilities regarding the state in which the quantum system will settle.
  - The real part of the complex number,
  - The imaginary part.
2. This is the integer representation of the qubits states. For example $\vert 16 \rangle =\vert 000000000000010000 \rangle$. In this simulation, the ancilla qubits will appear in this number.
3. These are the calculated probabilities of the qubit states by making use of the amplitude in 1.
4. These are the qubits being defined in the quantum register. In contrast to 2, this is the binary representation of the qubits. Ancilla qubits (if any), do not appear in this part of the result. This part also shows that the register width is the number of qubits.
  - Key qubits,
  - Plaintext qubits.

To summarize, the result in the shown example can be interpreted in the sense that the quantum register has only one state $\vert 000000000000010000 \rangle$ of probability of 1. All other states of the quantum system in the Hilbert space $\mathcal {H}^{\otimes 18}$, have 0 probability. From now on, only the quantum states and the associated probabilities are presented.

### QSDES functionality

In Table 2, the results of three arbitrary plaintext and keys of the classical SDES and QSDES are illustrated.

**Table 2:** QSDES functionality test

Plaintext	Classical		Quantum	
	Key	Ciphertext	Key and ciphertext	Probability
0001 0000	11 0001 0011	0011 0011	$\vert 11 0001 0011\,0011 0011 \rangle$	1
1110 1100	00 1110 1100	1110 0000	$\vert 00 1110 1100\,1110 0000 \rangle$	1
1011 0001	10 0111 1001	0001 1100	$\vert 10 0111 1001\,0001 1100 \rangle$	1

The resultant ciphertexts of the three arbitrary examples listed in Table 2 are identical for both classical and quantum platforms, which proves that the proposed QSDES works precisely as the classical SDES. Moreover, Table 3 shows the results of the QSDES when the key qubits are in superpositions. The plaintext (1001 1010) is encrypted simultaneously by all possible keys with only one query of QSDES, which is called natural parallelism. In Table 3, 1024 possibilities correspond to the key size, which is 10 qubits, are shown. Each state has a probability of $9.765623\times 10^{-4}$.

**Table 3:** QSDES results when key is in superposition

	Plaintext	Key and ciphertext	Probability
0	1001 1010	$\vert 0000000000\,11111001 \rangle$	$9.765623\times 10^{-04}$
1	1001 1010	$\vert 0000000001\,01010001 \rangle$	$9.765623\times 10^{-04}$
2	1001 1010	$\vert 0000000010\,01101001 \rangle$	$9.765623\times 10^{-04}$
$\vdots$	$\vdots$	$\vdots$	$\vdots$
1022	1001 1010	$\vert 1111111110\,11100110 \rangle$	$9.765623\times 10^{-04}$
1023	1001 1010	$\vert 1111111111\,00001011 \rangle$	$9.765623\times 10^{-04}$

### Quantum exhaustive key search

According to Eq. 5 in third section, the number of needed queries (Grover iterations) to find the secret key is calculated as follows:

14 $$\begin{aligned} R&=\frac{\pi }{4}\sqrt{N}\nonumber \\&=\frac{\pi }{4}\sqrt{1024}\nonumber \\&\approx 25.13 \end{aligned}$$

Table 4 illustrates the results of the quantum exhaustive search for the encryption key that used to encrypt the plaintext 0001 0000 and produced the ciphertext 0011 0011 with 25 Grover iterations.

**Table 4:** Quantum exhaustive key search

	Ciphertext	Oracle qubit and key and plaintext	Probability
0	0011 0011	$\vert 1\,0000000000\,00010000 \rangle$	$5.266659\times 10^{-07}$
1	0011 0011	$\vert 1\,0000000001\,00010000 \rangle$	$5.266659\times 10^{-07}$
$\vdots$	$\vdots$	$\vdots$	$\vdots$
787	0011 0011	$\vert 1\,1100010011\,00010000 \rangle$	0.9994553
$\vdots$	$\vdots$	$\vdots$	$\vdots$
1022	0011 0011	$\vert 1\,1111111110\,00010000 \rangle$	$5.266659\times 10^{-07}$
1023	0011 0011	$\vert 1\,1111111111\,00010000 \rangle$	$5.266659\times 10^{-07}$

**Fig. 13:**
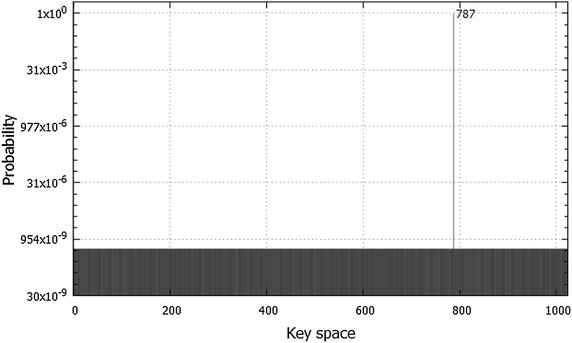
The probabilities of all possible keys for 10-bit key size. The keys are represented in decimal format. The chosen plaintext in this experiment is 00010000, and the ciphertext is 00110011. After 25 Grover iterations, the state 1100010011 (787 in decimal) is detected with probability of 0.9994553

Table 4 shows that the state $\vert 1\,1100010011\,0001000 \rangle$ has the highest probability 0.9994553, whereas all the other states have very low probabilities of $5.266659\times 10^{-07}$. Therefore, the secret key 11 0001 0011 is found in 25 queries in quantum compared to an average of 1023 queries in classical computing. The results of this experiment are illustrated in Fig. 13.

Surprisingly, the quantum attack was a highly competent in detecting the collision of multiple keys that can encrypt a particular plaintext and produce the same ciphertext. Consider $k_1\not = k_2$, but $SDES(k_1,P_i) = SDES(k_2,P_i)=C_i$. This kind of collision happens when the key length is larger than the plaintext length. On a classical computer, finding this type of collision is difficult whereas finding it on a quantum computer is easy. Furthermore, the existence of two or more keys that can encrypt a particular plaintext and produce the same ciphertext can make the quantum search much faster because multiple solutions or marked elements are available for Grover’s algorithm to search through. Table 5 presents the experimental results when two keys produce the same ciphertext.

**Table 5:** Quantum exhaustive key search when there are multiple solutions

	Ciphertext	Oracle qubit and key and plaintext	Probability
0	0011 0110	$\vert 1\,0000000000\,10100101 \rangle$	$4.118168\times 10^{-06}$
1	0011 0110	$\vert 1\,0000000001\,10100101 \rangle$	$4.118168\times 10^{-06}$
$\vdots$	$\vdots$	$\vdots$	$\vdots$
151	0011 0110	$\vert 1\,0010010111\,10100101 \rangle$	0.4978935
$\vdots$	$\vdots$	$\vdots$	$\vdots$
223	0011 0110	$\vert 1\,0011011111\,10100101 \rangle$	0.4978935
$\vdots$	$\vdots$	$\vdots$	$\vdots$
1022	0011 0110	$\vert 1\,1111111110\,10100101 \rangle$	$4.118168\times 10^{-06}$
1023	0011 0110	$\vert 1\,1111111111\,10100101 \rangle$	$4.118168\times 10^{-06}$

The quantum search in this experiment has been accomplished with only 18 Grover iterations. The number of iterations in case when there are two solutions ($M=2$), is calculated according to Eq. 6 in third section as follows:

15 $$\begin{aligned} R&=\frac{\pi }{4}\sqrt{\frac{N}{M}}\nonumber \\&=\frac{\pi }{4}\sqrt{\frac{1024}{2}}\nonumber \\&\approx 17.77 \end{aligned}$$

**Fig. 14:**
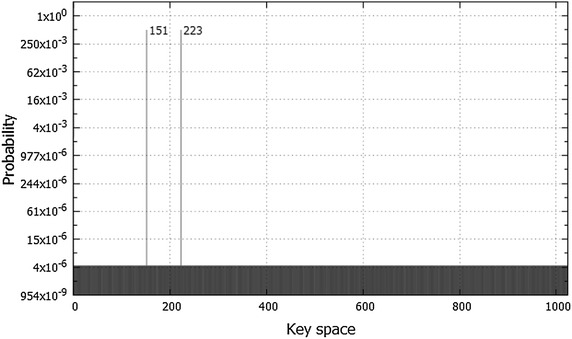
The probabilities of all possible keys for 10-bit key size. The keys are represented in decimal format. The chosen plaintext in this experiment is 10100101, and the ciphertext is 00110110. After 18 Grover iterations, the state 0010010111 (151 in decimal) and state 0011011111 (223 in decimal) are detected with probability of 0.4978935 each

As shown in Table 5, the chosen plaintext in the experiment is 1010 0101 and the corresponding ciphertext is 0011 0110. The table indicates that the two keys 0010010111 and 0011011111 have the highest probability of 0.4978935 each, whereas all other remaining states in the quantum register have a very low probability of $4.118168\times 10^{-06}$ each. Figure 14 illustrates the results of this experiment.

## Conclusion and future works

Quantum computing has rendered most of the classical asymmetric cryptosystems unsafe. However, the quantum threats to symmetric cryptosystems have not been investigated thoroughly compared with the asymmetric y cryptography. We claim that one of the reasons for the lack of studies on quantum cryptanalysis is that the symmetric algorithm must be implemented first on a quantum platform before the security strength of such a cryptosystem against any quantum attack can be evaluated. In this study, we proposed a method to fill the research gap between quantum computing and symmetric cryptography by presenting for the first time a quantum circuit for a classical symmetric cipher. The simplified DES cipher is used as a case study. The SDES is implemented on a quantum platform as a quantum circuit of a polynomial number of quantum gates. The entire study was tested on the quantum mechanics simulator libquantum. The functionality of the proposed design has been examined and proven by comparing the experimental results of the quantum SDES with that of the classical SDES. In addition, a quantum attack using Grover’s search algorithm has been conducted. The experimental results shows that the key can be recovered in $\frac{\pi }{4}\sqrt{N}$ computational steps.

The S-boxes of SDES and other ciphers are the most complicated components. In SDES, the S-boxes are statically predefined and implemented in this study as quantum circuits. The other types of S-boxes, specifically key-dependent dynamically generated ones, are interesting subjects to be investigated in the future.
